# Chassis optimization as a cornerstone for the application of synthetic biology based strategies in microbial secondary metabolism

**DOI:** 10.3389/fmicb.2015.00906

**Published:** 2015-09-09

**Authors:** Tiago Beites, Marta V. Mendes

**Affiliations:** ^1^I3S Instituto de Investigação e Inovação em Saúde, Universidade do PortoPorto, Portugal; ^2^Instituto de Biologia Molecular e Celular, Universidade do PortoPorto, Portugal

**Keywords:** chassis, heterologous expression, *Streptomyces*, specialized metabolites, genome streamlining, genome reduction

## Abstract

The increased number of bacterial genome sequencing projects has generated over the last years a large reservoir of genomic information. *In silico* analysis of this genomic data has renewed the interest in bacterial bioprospecting for bioactive compounds by unveiling novel biosynthetic gene clusters of unknown or uncharacterized metabolites. However, only a small fraction of those metabolites is produced under laboratory-controlled conditions; the remaining clusters represent a pool of novel metabolites that are waiting to be “awaken”. Activation of the biosynthetic gene clusters that present reduced or no expression (known as *cryptic* or *silent* clusters) by heterologous expression has emerged as a strategy for the identification and production of novel bioactive molecules. Synthetic biology, with engineering principles at its core, provides an excellent framework for the development of efficient heterologous systems for the expression of biosynthetic gene clusters. However, a common problem in its application is the host-interference problem, i.e., the unpredictable interactions between the device and the host that can hamper the desired output. Although an effort has been made to develop orthogonal devices, the most proficient way to overcome the host-interference problem is through genome simplification. In this review we present an overview on the strategies and tools used in the development of hosts/chassis for the heterologous expression of specialized metabolites biosynthetic gene clusters. Finally, we introduce the concept of specialized host as the next step of development of expression hosts.

## Introduction

Over the last century, specialized metabolites derived from microbial secondary metabolic pathways have been used by the pharmaceutical industry as a source of lead compounds to feed the drug discovery pipeline. However, in the past years there has been a decrease in the number of new drugs approved for clinical use, which is a reflection of a depleted drug discovery pipeline. In addition, natural products have been gradually dismissed due to defective bioprospecting programs that allocate a high number of resources but reach a very limited number of new compounds facing the so-called “dereplication” problem.

The development of high-throughput DNA sequencing technologies has rediscovered bacterial secondary metabolism as a reservoir of new bioactive compounds. The proliferation of genome sequencing projects (Harrison and Studholme, 2014) and the development of genome mining computational tools such as antiSMASH (Weber et al., 2015) have exposed bacterial genomes as the hosts of multiple secondary metabolites biosynthetic gene clusters (Nett et al., 2009). Presently there is a large pool of genomic information regarding biosynthetic gene clusters whose associated metabolites are unknown or uncharacterized, however, only a small fraction of those metabolites is produced under laboratory-controlled conditions. Activation of the biosynthetic gene clusters that present reduced or no expression (known as *cryptic* or *silent* clusters), has emerged as a key strategy for the identification and production of novel bioactive molecules (Ochi and Hosaka, 2012).

Among the several bacteria used as natural product factories (Gross and Loper, 2009; Weissman and Muller, 2010; Sansinenea and Ortiz, 2011; Mondol et al., 2013; Brito et al., 2015) the actinomycetes, in particular streptomycetes, stand out as the most prolific source of bioactive microbial metabolites (Berdy, 2005). Moreover, the vast (meta)genomic data collected during the post-genomic era and the identification of multiple cryptic secondary metabolite biosynthetic gene clusters in the genomes, has identified actinobacteria as a renewed source of novel natural products (Baltz, 2008). Due to *Streptomyces* known proficiency as natural producers, these bacteria have been widely used for the activation of cryptic gene clusters, namely by modulating the expression of genes coding for the cluster-situated regulators and/or biosynthetic proteins (Laureti et al., 2011; Olano et al., 2014). However, this strategy poses some technical challenges for many strains that have proven to be recalcitrant to genetic manipulation. To circumvent the resistance to genetic manipulation of natural producers, heterologous expression of secondary metabolite biosynthetic gene clusters has emerged as an alternative strategy for the activation of silent clusters. A paradigmatic example was the identification and characterization of 13 novel terpenes through the heterologous expression in *Streptomyces avermitilis* SUKA22 strain of 7 *Streptomyces*-derived genes annotated as terpene synthases (Yamada et al., 2015). Moreover, the continuous efforts to develop an extended *Streptomyces* molecular toolbox based on synthetic biology principles (Rudolph et al., 2013; Luo et al., 2015) has potentiated even more cryptic gene clusters as an alternative for the discovery of novel bioactive compounds (Shao et al., 2013).

In the context of microbial specialized metabolite heterologous production, the development of suitable expression host strains, or as it is called in the synthetic biology jargon, the chassis, is key for the use of synthetic biological systems to the production and characterization of new bioactive metabolites. In this review we will focus on the strategies for development of Actinobacteria cell platforms devoted to the production of small molecules derived from microbial secondary metabolism and review the current status on natural compound heterologous production.

## Genome Streamlining as a Way to Genome Simplification

The increasingly competitive and efficient new generation sequencing techniques have completely revolutionized biological sciences. The applications are diverse and impacted the way how biological systems are approached, i.e., instead of a gene-based approach it is now possible to face the cell as a whole through a genome-based approach. And it is even possible to go one level up and study a community of organisms through meta-genomics. However, the massive amount of data currently available provided a clearer picture on the extreme complexity that a single cell encloses, exposing our lack of understanding on how the different network components are wired. Furthermore, the sequencing of genomes revealed a high percentage of predicted proteins with no discernible localization and/or function (Ijaq et al., 2015).

Cell complexity poses many challenges to synthetic biology, since one should not expect that the synthetic device would not interact with endogenous components of the host cell. In fact, the complexity of the system exponentially increases possibilities of unpredictable interactions that may hamper the output. As a response to this limitation, an effort has been made towards the generation of orthologous systems (Channon et al., 2008). Orthogonality is a concept borrowed from mathematics and computer science and it refers to synthetic devices that work independently from the host, meaning that the interference will be minimal. The engineering of ribosomes that recognize a different genetic code is a good example on the efforts that have been made toward orthogonal systems (Wang et al., 2007). However, even in this case the activity of the ribosomes is still very dependent on the cellular machinery and prone to be modulated by different factors (de las Heras et al., 2008).

The rationale behind genome streamlining aims at solving this host interference problem by reducing the complexity of the chassis genome (Leprince et al., 2012b). However, although genome simplification is a very logical step as a general concept, still there are many questions to answer. What strains will be used? What regions should be deleted? How should genome streamlining be performed? Where is the threshold beyond which genome simplification does not bring any additional advantages? The answer to these questions is highly dependent on the downstream application. In fact, as it will be discussed in the next sections, there is a vast multitude of genome streamlining workflows that can be used to meet the different aims.

Nevertheless, there is a primal question to all genome streamlining projects: what makes a good chassis? In spite of the unavoidable specificities, one could point out four main characteristics that must be taken into account: genetic manageability, growth robustness, genetic stability and the ability to accurately predict interactions between the synthetic device and the chassis. In the particular case of chassis development for the production of microbial specialized metabolites, one could add that it should also possess a minimal extracellular metabolome profile that would simplify the purification of the desirable molecule (Gomez-Escribano and Bibb, 2011).

The clarification of the minimal genome and reduced genome concepts is also of great importance when it comes to genome streamlining. The quest for the minimal genome envisioned the determination of the minimal set of genes necessary to sustain life (de Lorenzo, 2011). The versions of the minimal genome have been swinging in number and components with no apparent consensus, mainly due to environmental constraints, i.e., different environments equal different requirements (Danchin, 2012). Nevertheless, these studies are dealing with the limits of life and thereby of paramount relevance to basic biological questions such as the identification of the “core” genome. The “core” genome defined as the set of common essential genes, is a concept intimately linked to the development of minimal or reduced genomes. For instance, comparative genome analysis of 17 genomes led to the definition of *Streptomyces* core genome composed of 2018 orthologous genes that corresponded to 24–38% of the analyzed genomes (Kim et al., 2015). However, if one intends to develop a chassis for biotechnological downstream applications, the suitability and applicability of such minimal platforms is not clear. In particular, if we consider the heterologous expression of specialized metabolites, the efficient supply of precursor units requires genetic features that go beyond the core genome. Therefore, concerning the genome streamlining for the heterologous expression of microbial specialized metabolites the reduced genome concept seems more suitable than the minimal genome.

## Genome Editing Toolkit

Homologous recombination has long been used to genetically manipulate strains, getting advantage of the natural recombination systems. However, relying solely on the endogenous machinery may constitute a low efficient approach in some cases (Komatsu et al., 2010). Systems like the bacteriophage-derived -Red recombinase were shown to greatly increase the efficiency of homologous recombination and thereby very useful for genome editing (Murphy, 1998). This system only requires a minimum of 30–50 bp overlapping flanking regions, which attests its efficiency (Karlinsey, 2007). Recently, a system based on the meganuclease I-SecI from *Sacharomyces cereviseae* was developed for the genetic manipulation of actinomycetes (Fernandez-Martinez and Bibb, 2014). The strategy relies on the introduction of a DNA break by the endonuclease at an unique 18 bp recognition sequence that can only be repaired by homologous recombination. The double recombinants recovery efficiency reported (27–52%) validates this new system as a valuable tool for genome editing, particularly if we consider the traditional low-number of double recombinants obtained in the *Streptomyces* field (Kieser et al., 2000).

Interestingly, recombineering without the action of a recombinase was shown to be possible in some Gram-negative bacteria. In this case the transformation of cells with synthetic single-stranded DNA (ssDNA) oligonucleotides was shown to be able to recombine with genomic DNA – a process denominated by oligonucleotide recombineering (Swingle et al., 2010). All homologous recombination reactions rely on a ssDNA intermediate that will pair with the complementary strand in the target double-stranded DNA (dsDNA). The work of Li et al. (2013) has shown that the ssDNA is incorporated during DNA replication as an Okazaki fragment, demonstrating the importance of DNA polymerase in this process.

In a more high-throughput manner, Wang et al. (2009) developed a system that aims at editing multiple locus applying the oligonucleotide recombineering concept – Multiplex Automated Genome Engineering (MAGE). The MAGE technology is an automated process that transforms multiple oligonucleotides into bacteria (*Escherichia coli* was used as the working model) in an iterative way. The application of multiple cycles of transformation allows the formation of a heterogeneous population, which enables the usage of directed evolution to develop strains with interesting characteristics (Wang et al., 2009).

Site-specific recombinases catalyze the recombination between two specific DNA sequences, performing all the reactions needed namely DNA excision, inversion, integration, and translocation. Genome editing based on these enzymes has proven to be a very efficient tool (Branda and Dymecki, 2004). Most systems currently in use are based on the Cre/*loxP* from the P1 phage and Flp/FRT from yeast (Schweizer, 2003; Branda and Dymecki, 2004). Both Cre and Flp are tyrosine recombinases that recognize 34 bp target sites – *loxP* and FRT, respectively – and catalyze the site-specific recombination event. Depending on the orientation of the target sites, Cre and Flp can either promote excision (direct repeats) or inversion (inverted repeats; for a detailed review on recombinases see Siegl and Luzhetskyy, 2012). Interestingly, the excision ability of these enzymes allow the generation of marker-less mutant strains, which contributes to a more predictable phenotype and facilitates further genetic manipulations (Khodakaramian et al., 2006).

The fact that none of these two recombinases need a co-factor provided by the host, gives this system a wide applicability (Kuhn and Torres, 2002). However, some limitations may be encountered, such as codon usage. This is a common problem, for example, in heterologous gene expression in high GC content organisms such as Actinobacteria. To circumvent codon usage issues, Herrmann et al. (2012) synthesized Cre and Flp versions with codons compatible with Actinobacteria usage and demonstrated its high efficiency and accuracy.

The Clustered Regularly Interspaced Short Palindromic Repeats (CRISPRs)/CRISPR associated (Cas) proteins belong to the so-called bacterial adaptive immune system (Haft et al., 2005). The different CRISPR systems can be grouped in three different systems. While type I and type III are characterized by requiring multiple Cas proteins to induce the cleavage of their target DNA, type II systems only require the Cas9 endonuclease (Haft et al., 2005). The versatility of Cas9 allows it to introduce double strand breaks in a target genomic sequence through the co-expression of customized single guide RNA (Jinek et al., 2012). This characteristic makes CRISPR-Cas9 a very attractive tool for genome editing. Putting a focus on secondary metabolism, this system was already successfully applied in the deletion of two genes from the actinorhodin biosynthetic gene cluster in *S. coelicolor* (Tong et al., 2015).

## Top–down Strategies for Genome Streamlining

**FIGURE 1:**
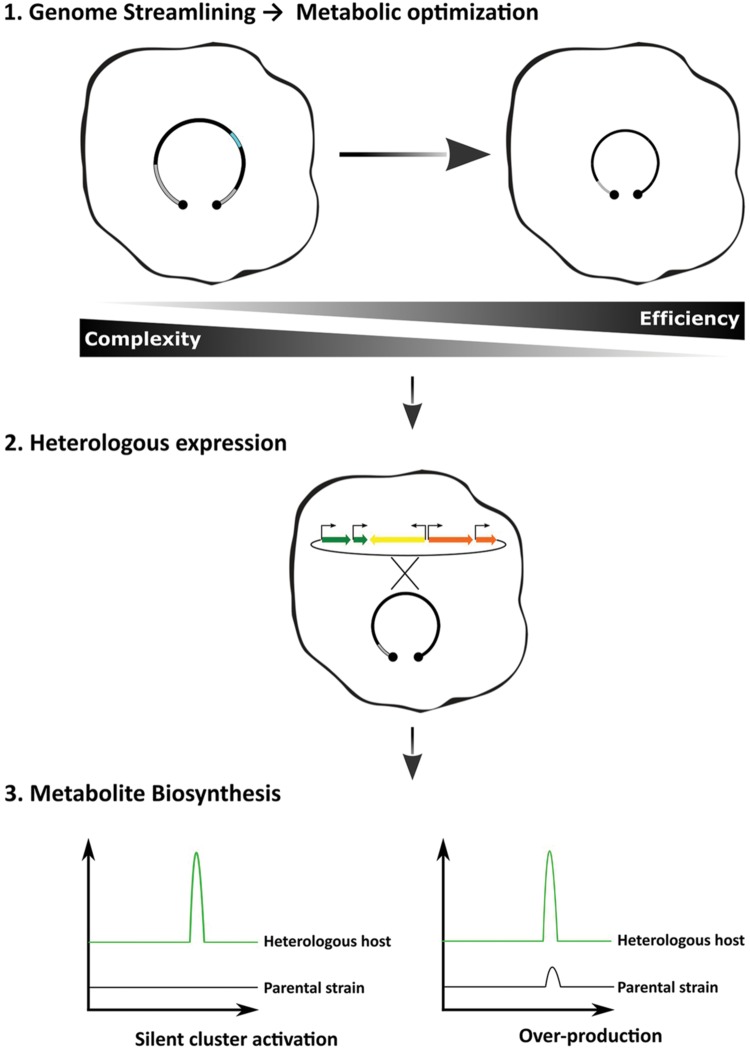
**Optimized workflow for the heterologous expression of biosynthetic gene clusters.** Applying the concept of genome simplification, one should expect that the host-interference problem would be minimized rendering more efficient strains. The methods of gene delivery and cluster refactoring can also improve the desired outcome, i.e., strains combining the ability to produce heterologous cryptic compounds and to behave as over-producers.

From a biotechnological point of view, top–down strategies are the most attractive, because they consist in the reduction of existing genomes. By deleting parts that are predicted to be non-essential to the microorganisms, or parts that may contribute negatively to the pretended outcome, it is expected to obtain more efficient and tractable chassis. Indeed, genome reduction by the deletion of non-essential genes has resulted in increased genome stability, growth robustness, simplification of the secreted metabolome and increased availability of precursor units (Gomez-Escribano and Bibb, 2011; Zhou et al., 2012; Komatsu et al., 2013). Genome reduction as the first step in the heterologous expression workflow is expected to render strains that will not only be capable of producing cryptic specialized metabolites, but also to over-produce the final product (**Figure 1**).

Efforts to streamline genomes have been particularly incident in *Bacillus subtilis* and in the molecular biology “workhorse” *E. coli*, mainly using directed mutagenesis strategies, such as site-specific recombination (Juhas et al., 2014). Multiple reports on genome reduction of industrial relevant organisms show a maximum reduction level below 25% of the total genome: *E. coli*, 15.3% (Posfai et al., 2006); *B. subtillis*, 20.7% (Morimoto et al., 2008); *S. avermitilis*, 16.9–18.6% (Komatsu et al., 2010) and *Corynebacterium glutamicum* 22% (Unthan et al., 2015). These low reduction percentages highlight how we are still very far from knowing how the networks that compose life are wired. In spite of these limitations, the application of directed mutagenesis to genome streamlining rendered some strains with interesting properties for biotechnological applications such as *Streptomyces*-derived strains, where genome reduction allowed the heterologous expression and production of small molecules (**Table 1**).

**Table 1:** Production yields of specialized metabolites heterologously expressed in *Streptomyces*-based chassis and in the native producers.

Compound	Metabolite class	Heterologous expression yield (mg/L)	Natural producer	Native expression yield (mg/L)	Reference
***Streptomyces coelicolor M512 (M145** Δ***redD***Δ***actII-ORF4*****)***					Floriano and Bibb, 1996
Clorobiocin	Aminocoumarin	26	*S. roseochromogenes* var. *oscitans* DS 12.976	25	Eustaquio et al., 2005
Coumermycin A1	Aminocoumarin	7	*S. rishiriensis* DSM 40489	5	Wolpert et al., 2008
Novobiocin	Aminocoumarin	20–42	*S. spheroides NCIMB 11891*	30–40	Eustaquio et al., 2005
***S. coelicolor*** **M1146 (M145** Δ***act***Δ***red***Δ***cpk***Δ***cda*****)**					Gomez-Escribano and Bibb, 2011
Cephamycin C	NRP	NP	*S. clavuligerus* ATCC 27064	3640	Martinez-Burgo et al., 2014
Chloramphenicol	Shikimate derived metabolite	40	*S. venezuelae* ATCC 10712	20	Gomez-Escribano and Bibb, 2011; Komatsu et al., 2013
GE2270	Thiopeptide	1–3	*Planobispora rosea* ATCC 53733	200	Tocchetti et al., 2013; Flinspach et al., 2014
***S. coelicolor*** **M1152 (M1146** ***rpoB[C1298T])***					Gomez-Escribano and Bibb, 2011
Tacrolimus	PK-I	2.81	*S. tsukubaensis* NRRL 18488	19	Martinez-Castro et al., 2013; Jones et al., 2013
***S. avermitilis*** **SUKA17/SUKA22 (**Δ**(79455-1595563 nt)::*****loxP*** Δ**(*****olmA4*****-*****olmC*****)::mut-*****loxP*** Δ**terpenes)**					Komatsu et al., 2010, 2013
Bafilomycin B1	PK-I	16	*Kitasatospora setae* KM-6054	3.5	Komatsu et al., 2013
Cephamycin C	NRP	85	*S. clavuligerus* ATCC 27064	3640	Komatsu et al., 2010; Martinez-Burgo et al., 2014
Chloramphenicol	Shikimate derived metabolite	250	*S. venezuelae* ATCC 10712	20	Komatsu et al., 2013
Clavulanic acid	Peptide	16	*S. clavuligerus* ATCC 27064	120–135	Li and Townsend, 2006; Komatsu et al., 2010
Erythromycin	PK-I	4	*Saccharopolyspora erythraea* NRRL 2338	120–200	Peano et al., 2007, 2012; Komatsu et al., 2013
Holomycin	NRP	8	*S. clavuligerus* ATCC 27064	<1	Komatsu et al., 2013
Lactacystin	NRP-PK	30	*S. lactacystinaeus* OM-6519	2	Komatsu et al., 2013
Leptomycin	PK-I	5	*Streptomyces* sp. ATS1287	30–38	Hamamoto et al., 1983; Komatsu et al., 2013
Nemadectin	PK-I	1.6	*S. cyaneogriseus subsp. noncyanogenus* NRRL 15774	310	Tsou et al., 1989; Komatsu et al., 2013
Oxytetracycline	PK-II	20	*S. rimosus NRRL 2234*	38.5	Komatsu et al., 2013
Pholipomycin	Phosphoglycolipid	20	*S. clavuligerus* ATCC 27064	NP – Cryptic	Komatsu et al., 2013
Ribostamycin	Aminoglycoside	8	*S. ribosidificus* ATCC 21294	185	Komatsu et al., 2013
Shinorine	Mycosporin	154	*Actinosynnema mirum* DSM 43827	NP – Cryptic	Miyamoto et al., 2014
***S. lividans*** **TK24 (str-6 SLP2**^**-**^ **SLP3**^**-**^**)**					
Daptomycin	Lipopeptide	55	*S. roseosporus*	150–1000	Penn et al., 2006
GE37468	Thiopeptide	2–3	*Streptomyces* sp. ATCC 55365	6–8	Young and Walsh, 2011

*NRP, non-ribosomal peptide; PK, polyketide; NP, no production detected.*

Although successful at some extent, directed mutagenesis still has major limitations when it comes to streamlining genomes, mainly due to the lack of knowledge regarding cell components and, more importantly, their interactions. As counterintuitive as it may seems, a random mutation approach may be a good alternative for genome streamlining. The creation of libraries of mutants with random deletions and the selection of strains with desirable traits gives a “Darwinian evolution” twist to the process, by combining unbiased mutations with a selective pressure toward a certain outcome.

This approach has been successfully applied to *Pseudomanas putida* (Leprince et al., 2012a). Two mini-transposons – mini-Tn*5 KpF* and mini-Tn*5 TF* – were modified in order to contain selective markers and a FRT site (recognized by Flt recombinase). The isolation of mutants containing both transposon insertions in random genome locations and the usage of the recombinase Flt allowed the deletion of certain segments of the chromosome and the selection of derived strains with unaffected fitness. Single segment deleted mutants reached a genome reduction of 4.1%. But perhaps the most interesting feature of this study is the capacity of iteration. Indeed a second cycle of deletion allowed the isolation of strains with a maximum of 7.4% genome reduction (Leprince et al., 2012a). In a short-term perspective this kind of approaches may constitute the more efficient way to streamline a genome. Furthermore, the creation of mutant libraries is also interesting due to the possibility of choosing different strains for different purposes, which might be very advantageous to biotechnology.

The usage of MAGE applied to the optimization of the production of 1-deoxy-D-xylulose-5-phosphate (DXP) – an industrially important isoprenoid lycopene – in *E. coli* was proven to be successful (Wang et al., 2009). The authors targeted 24 genes involved in the biosynthesis of DXP and obtained a very heterogeneous mutant population, from which some isolated strains revealed a fivefold increase in DXP yields (Wang et al., 2009). Although the oligonucleotides targeted pre-defined genes, this technique should be regarded as random mutagenesis due to the unpredictable combinatorial mutations that render the final heterogeneous population. This way, one can envision MAGE technology as a powerful technique to streamline genomes toward the optimization of a chassis.

The usage of random mutagenesis and isolation of mutants with interesting phenotypes is also at the core of strain development in the pharmaceutical industry. This actually means that genome streamlining has been practiced for decades now, although with different purposes than obtaining a suitable chassis for expressing synthetic pathways. Nevertheless, the metabolic network of these strains is highly optimized for the production of a certain product and may actually be useful for the heterologous expression of secondary metabolite biosynthetic gene clusters. *S. ambofaciens* is a natural producer of the important polyketide spiramycin. BES2074 strain derived from the spiramycin over-producer 111–59 was isolated and shown to have a blockage in the production of spiramycin (Richardson et al., 1990). The introduction of a BAC vector with the entire cyclic lipopeptide A54145 biosynthetic gene cluster rendered a final yield that was 285% higher than its natural producer *S. fradiae* (Alexander et al., 2010).

## *Streptomyces*-Based Expression Hosts

The natural ability of *Streptomyces* bacteria to produce a wide range of specialized metabolites grants them, in principle, the metabolic background needed for the heterologous expression of biosynthetic gene clusters. Thus, it is not surprising that *Streptomyces* bacteria have been frequently used for the development of optimized expression hosts.

*Streptomyces avermitilis* is a producer of the anti-infective avermectin with a linear chromosome of 9.02 Mb (Omura et al., 2001). Taking advantage of the typical *Streptomyces* genome organization, a region from the left subtelomeric region (∼2 Mb) that corresponds to the more variable genome regions was deleted using general homologous recombination or site-specific recombination (Cre-*loxP*) techniques. A series of genome-reduced *S. avermitilis* mutants strains were obtained of which we should highlight SUKA5 and SUKA22 (isogenic to SUKA17) strains that had a genome reduction of 17.9 and 18.5% respectively, and a 78% reduction of the total transposase genes when compared to the wild-type (Komatsu et al., 2010). *S. avermitilis* SUKA5 strain had the oligomycin biosynthetic gene cluster deleted in addition to the left subtelomeric region that included avermectin and filipin biosynthetic gene clusters; SUKA22 strain was a derivative of SUKA5 that had the terpene biosynthetic encoding genes deleted (Komatsu et al., 2010). In both strains, growth was not significantly affected and no production of endogenous metabolites was observed (Komatsu et al., 2013). The generated strains presented advantages for the heterologous expression of biosynthetic gene clusters, presumably due to the lack of endogenous metabolic pathways that would compete for cell resources and to a decrease in genome instability. The authors were able to express heterologously several biosynthetic gene clusters (**Table 1**) (Komatsu et al., 2010, 2013) including the cryptic biosynthetic clusters of pholipomycin from *S. clavuligerus* ATCC 27064 and shironine from *Actinosynnema mirum* DSM 43827. In most of the cases, the streamlined strains produced higher titers of the compounds in comparison with the natural producers (**Table 1**).

Although not originally generated for the heterologous expression of specialized metabolites, *S. coelicolor* M512 (Floriano and Bibb, 1996) that lacks the cluster-situated regulators of the actinorhodin and undecylprodigiosin biosynthetic gene clusters, was used successfully in the expression of clorobiocin, coumermycin, and novobiocin among others (**Table 1**). Few years later, *S. coelicolor* was subjected to a rational genome reduction. The streamlining project of *S. coelicolor* M145 strain (derivative of *S. coelicolor* A3(2) that lacks the two natural plasmids SCP1 and SCP2) was not as drastic as the *S. avermitilis* project, since it only targeted the native secondary metabolite biosynthetic gene clusters (Gomez-Escribano and Bibb, 2011). In this case the biosynthetic gene clusters of the secondary metabolites majorly produced by this strain (actinorhodin, prodiginine, CPK and CDA) were sequentially deleted by homologous double-recombination, generating a plethora of streamlined strains characterized by a low percentage of genome reduction (2%). Interestingly, the authors did not solely proceed with genome reduction. In fact, a strain optimization was putted in place through the introduction of point mutations into *rpoB* and *rpsL* with the expectation of pleiotropically increasing the production of secondary metabolites. The authors succeeded to heterologously express the chloramphenicol and congocidine gene clusters from *S. venezuelae* ATCC 10712 and *S. ambofaciens* ATCC 23877 (Gomez-Escribano and Bibb, 2011).

Other group also streamlined *S. coelicolor* M145 genome by sequential deletion of all the gene clusters containing polyketide synthases (PKS) and non-ribosomal protein synthases (NRPS), as well as a 900 kb fragment from a sub-telomeric region (Zhou et al., 2012). A double homologous recombination strategy was followed in this work. To attest the usefulness of the streamlined strains, the authors overexpressed the actinorhodin biosynthetic gene cluster. The results have shown that the strains with a reduced genome produced higher titers of actinorhodin. In this case the strategy was focused on the production of polyketides and the fact that the metabolism of *S. coelicolor* is naturally optimized to produce this kind of compounds, together with the lack of metabolic pathways competing for the same precursor molecules, accounts for the observed over-producing phenotypes.

These examples, especially in the case of *S. avermitilis*, foresee the ability to use these strains as universal chassis for the production of secondary metabolites. However, one should not forget that all these strains are still naturally constrained by their genomic background. In fact, the biological complexity that is still present in these strains and the lack of true orthogonal systems are likely to hamper their universality and application in the context of heterologous expression at the industrial scale. This is attested by the different production yields obtained for the same specialized metabolite (**Table 1**). Although the expression of the chloramphenicol gene cluster in *S. coelicolor* M1146 (Gomez-Escribano and Bibb, 2011) and *S. avermitilis* SUKA22 (Komatsu et al., 2013) led to higher production levels than the native producer *S. venezuelae* ATCC 10712, *S. avermitilis* SUKA22 was able to produce sixfold higher amounts than *S. coelicolor* M1146. Conversely, heterologous expression of the cephamycin C gene cluster in the same strains resulted in no production or lower yields than in the native producer *S. clavuligerus* (Komatsu et al., 2013; Martinez-Burgo et al., 2014) (**Table 1**). Thus, a more practical alternative to develop expression chassis would be to use strains that have been previously optimized to produce a certain class of compounds such as the strains already in use in the industry. These strains not only possess an optimized metabolism, but also have been adapted to the industrial process through the improvement of other characteristics, such as morphology (Nieminen et al., 2013). A posterior genome streamline process of these strains would further optimize their ability to perform as “specialized chassis.”

## Bottom–up Strategies for Genome Streamlining

The bottom–up strategy deals with the design and development of strains from the scratch. Regarding chassis construction with biotechnological purposes the bottom–up strategy would really put engineering principles at the core of the technology as it is preconized in the synthetic biology field. In the future, cells designed to deliver a certain function could be developed through the wiring of synthetic metabolic pathways, opening endless possibilities. However, this is not a realistic scenario yet. In fact, the efforts made in this field are more related with understanding more basic questions, such as the origin of life (Leprince et al., 2012b).

The current vision on bottom–up approaches encompasses the assembly of DNA along with core protein machinery encapsulated on vesicles, allowing self-replication and energy generation (Kurihara et al., 2011). Some efforts done within the synthetic virology field can also be included in the bottom–up category. Due to their small genomes and low requirements for replication, the molecular networks enclosed in a virus are more discernible. Thus, the design or re-factoring of a virus is an achievable goal with our technology and with possible interesting applications, such as gene delivery (Guenther et al., 2014). Although it was still not possible to make functional “blueprint” for life, a landmark on bottom–up strategies was achieved when a total synthetic genome of *Mycoplasma mycoides* was assembled and successfully transplanted into a *M. capricolum* host devoid of its own genome (Gibson et al., 2010).

## Naturally Optimized Genomes

From a pragmatic point of view, one could consider using strains that went through a natural genome streamlining process and use them as a chassis. Although there might be a lack of knowledge in the genetic circuitries of these strains and thus being a sub-optimal strategy, it may be useful in some cases.

Regarding secondary metabolite biosynthesis, *S. albus* J1074 is a good example of a suitable chassis for heterologous expression of biosynthetic gene clusters with a naturally streamlined genome. This strain possesses the smallest genome in *Streptomyces* genus (∼6.8 Mb) and a low number of gene duplications (Zaburannyi et al., 2014). This highlights the tendency of redundancy reduction in this strain, which constitutes a good characteristic for a chassis. A transcriptomic analysis showed an early metabolic switch, which is coherent with the high growth rate presented by this strain (Zaburannyi et al., 2014). In addition it is known that this strain can be manipulated genetically in a very efficient way. Altogether these characteristics allowed its usage as an efficient chassis for heterologous biosynthetic gene cluster expression and, more importantly, a flexible one. The anti-tumor anthracycline steffimycin biosynthetic gene cluster from *S. steffisburgensis* (Gullon et al., 2006), fredericamycin from *S. griseus* (Wendt-Pienkowski et al., 2005), napyradiomycin from *S. aculeolatus* (Winter et al., 2007) and cyclooctatin from *S. melanosporofaciens* (Kim et al., 2009) were all cloned and successfully expressed in *S. albus.*

## Conclusion and Future Perspectives

The identification of the so-called *silent* or *cryptic* clusters shows that under laboratory conditions bacteria are not able to present the metabolic flexibility needed for the production of all encoded metabolites. The development of “*universal” Streptomyces* heterologous expression hosts based on genetically modified strains of *Streptomyces* sp. (Komatsu et al., 2010, 2013; Gomez-Escribano and Bibb, 2011; Zhou et al., 2012; Ikeda et al., 2013) has validated the heterologous expression of secondary metabolite clusters particularly for the activation of silent clusters and characterization of new metabolites. However, the production yields are generally low and often lower than the original producers (**Table 1**) unveiling a deficient metabolic flux background and undermining this strategy for industrial applications that rely on high production yields.

For an industrial downstream application of the heterologous expression of specialized metabolites, there is the need of combining the stability features of the universal expression hosts with the metabolic fitness for producing high-added value products of the industrial strains. The vast knowledge regarding *Streptomyces* metabolic networks and their regulation (Liu et al., 2013) as well as a successful history on secondary metabolism engineering (Olano et al., 2008) turns *Streptomyces*, particularly those strains already optimized for industrial processes, in attractive subjects for the development of *metabolite class specialized hosts* through the synergic use of synthetic biology, system biology and metabolic engineering methodologies.

## Conflict of Interest Statement

The authors declare that the research was conducted in the absence of any commercial or financial relationships that could be construed as a potential conflict of interest.
